# Fabrication and Dielectric Validation of an Arm Phantom for Electromyostimulation

**DOI:** 10.3390/bioengineering11070724

**Published:** 2024-07-17

**Authors:** Katja Uhrhan, Esther Schwindt, Hartmut Witte

**Affiliations:** 1 Biomechatronics Group, Department of Mechanical Engineering, Technische Universität Ilmenau, 98693 Ilmenau, Germany; hartmut.witte@tu-ilmenau.de; 2 Ostbayerische Technische Hochschule Regensburg, 93053 Regensburg, Germany; esther.schwindt@st.oth-regensburg.de

**Keywords:** tissue phantom, electromyostimulation, electrical muscle stimulation, dielectric validation, electromyography

## Abstract

Electromyostimulation (EMS) is an up-and-coming training method that demands further fundamental research regarding its safety and efficacy. To investigate the influence of different stimulation parameters, electrode positions and electrode sizes on the resulting voltage in the tissue, a tissue mimicking phantom is needed. Therefore, this study describes the fabrication of a hydrogel arm phantom for EMS applications with the tissue layers of skin, fat, blood and muscle. The phantom was dielectrically validated in the frequency range of 20 Hz to 100 Hz. We also conducted electromyography (EMG) recordings during EMS on the phantom and compared them with the same measurements on a human arm. The phantom reproduces the dielectric properties of the tissues with deviations ranging from 0.8% to more than 100%. Although we found it difficult to find a compromise between mimicking the permittivity and electrical conductivity at the same time, the EMS–EMG measurements showed similar waveforms (1.9–9.5% deviation) in the phantom and human. Our research contributes to the field of dielectric tissue phantoms, as it proposes a multilayer arm phantom for EMS applications. Consequently, the phantom can be used for initial EMS investigations, but future research should focus on further improving the dielectric properties.

## 1. Introduction

Electromyostimulation (EMS) is a relatively new training method in which skeletal muscles contract due to external electrical impulses. One can differentiate between whole-body EMS (WB-EMS), in which current is applied simultaneously via at least six channels involving all major muscle groups, and single-muscle EMS, where a pair of electrodes is applied specifically to one or two target muscles [1]. The advantages of WB-EMS are lower joint load and time efficiency [1], while local EMS can be used for rehabilitation purposes [2] or to compensate for muscular imbalances by a targeted activation of the weaker side [1]. However, because of its unnatural recruitment of motor units, EMS carries a high potential for muscle damage [3], especially when applied for the first time [4]. Therefore, an individualized setting of stimulation parameters is essential in order to adequately stress the muscles. The amount of voltage reaching the muscle depends on various factors such as tissue composition, skin resistance, stimulation parameters, electrode configuration and electrode size [5,6]. In practice, however, the stimulation settings are currently only selected based on the subjective perception of the exerciser and the experience of the trainer [1]. In order to investigate the influence of the above-mentioned factors in more detail and to incorporate them into training control, a phantom for EMS applications that mimics the dielectric properties of biological tissue in the relevant frequency range is necessary. In this way, the influence of different electrode positions, stimulation parameters and tissue compositions on the voltage distribution within the tissue could be investigated without the ethical concerns of studies on animals or humans. Studies of this kind could make an important contribution to a better understanding of EMS, and thus increase the safety and efficacy of the training method.

In the biomedical field, phantoms are commonly used for research and educational purposes. The majority of these phantoms are used in radiological imaging applications [7,8,9], for medical training [10,11,12] or to test medical devices [13,14]. While the aforementioned applications mainly focus on mimicking mechanical, acoustic or optical properties of biological tissue, fewer phantoms exist that simulate the dielectric properties. One of these phantoms is presented in Aminzadeh et al. [15], which simulates the dielectric properties of skin and muscle in the microwave and millimeter-wave range. It was designed for frequencies from 26.5 GHz to 40 GHz, using low-cost fabrication materials such as distilled water, agar, polyethylene, guar gum and gelatin. The phantom reproduces the dielectric properties of the tissue with a maximum deviation of 10%. 

Costanzo et al. [16] developed a multilayer human arm phantom for microwave body sensor applications. They fabricated different tissue layers (skin, muscle, blood, fat) with different compositions of water, gelatin, oil, soap and salt. They adapted the dielectric properties in the frequency range of 500 MHz to 5 GHz and achieved maximum deviations of 10% for permittivity and 36% for electrical conductivity compared to biological tissue. Similar research was performed by Khorshid et al. [17], who developed a layered arm phantom for intra-body communication applications in the frequency range of 100 kHz to 100 MHz. They produced oil-based samples mimicking the dielectric properties of skin, fat, muscle, cortical bone and bone marrow. 

The research of Toyoda et al. [18] describes a hydrogel-based phantom to which antimony-doped tin oxide (ATO/TiO_2_) was added. The phantom mimics the dielectric properties of muscle tissue in the frequency range of 100 kHz to 1 MHz. Similar research was conducted by Anand et al. [19], who fabricated a hydrogel forearm phantom to mimic the dielectric properties of muscle, fat and blood from 1 kHz to 2 MHz.

While the above-mentioned phantoms focus on mimicking the conductivity and permittivity of specific tissue types, they are not specifically designed for EMS applications, and thus only apply to frequencies that lie far above the frequency range of EMS, which mainly ranges from 1 Hz to 150 Hz. The research of Fukuhara et al. [5] is one of the few publications that addresses this low-frequency range. They propose a fat and muscle phantom for EMS investigations using glycerol, polyethylene powder, agar and sodium chloride. However, it is a very simplified phantom composed of only two tissue types.

Goyal et al. [20] presented a skin phantom in the range of 1–1000 Hz, which consists of a layer of polyvinyl alcohol cryogel with saline solution and a second layer of polydimethylsiloxane, carbon black and barium titanate. In addition to the more complex fabrication and more expensive ingredients compared to gelatin hydrogel phantoms, the phantom is limited to only skin tissue. The research of Kalra et al. [21] and Yu et al. [22] also cover the low-frequency range used for EMS but as well focus on just one tissue type each, and hence do not represent an entire body part. 

Consequently, few dielectric tissue phantoms have been presented for the low-frequency range, and none of them have been especially developed for extensive EMS examinations including more than two tissue layers. Therefore, we describe a low-cost fabrication of an upper arm phantom for EMS investigations with the relevant tissue layers of skin, fat, blood and muscle. Subsequently, the developed phantom is validated regarding its ability to mimic the dielectric properties of the different tissue types and its suitability for EMS examinations. For this purpose, we first measured the dielectric properties of the individual phantom layers using the parallel plate method and compared the results with reference values from the IT’IS Foundation tissue database [23]. Subsequently, we conducted electromyography (EMG) during EMS on the phantom and validated the recorded EMS signals with the same measurements on a human’s upper arm. 

## 2. Materials and Methods

### 2.1. Phantom Realization Procedure

To fabricate the phantom samples mimicking the dielectric properties of skin, fat, blood and muscle, we used inexpensive customary materials such as gelatin (“Blatt-Gelatine weiß”, K-CLASSIC, Neckarsulm, Germany; “Gelatine Pulver”, Naturix24, Dransfeld, Germany), distilled water (“Destilliertes Wasser”, Priva, Hamburg, Germany), sunflower oil (“Reines Sonnenblumenöl”, VitaD’or, Hilter, Germany), dishwashing detergent (“Spülmittel Aloe Vera”, W5, Stolberg, Germany) and iodized table salt (“AlpenJodSalz”, Bad Reichenhaller, Heilbronn, Germany). We prepared four to six samples with different material compositions for each phantom type, using mixing ratios from the literature [16,21,22] as a guide and modifying them according to the dielectric measurements we conducted. 

Table 1 provides an overview of the material concentrations of the various phantom samples. To initially characterize the samples, the mixture was poured into Petri dishes with a diameter of 850 mm. Once the appropriate mixing ratios had been identified, a larger casting container (33 cm × 9 cm base area) was used to produce the arm phantom. Regardless of the size of the samples, the phantoms were prepared as follows: First, the gelatin sheets were soaked in distilled water for ten minutes, while the other ingredients, such as oil, dishwashing detergent, distilled water and salt, were already mixed together in the selected quantities. The soaking step can be skipped when using gelatin powder. The mixture, still without gelatin, was heated to 70 °C with gentle stirring [16]. Once the mixture reached the required temperature, it was removed from the heating plate. Then, the squeezed-out gelatin sheets were stirred into the mixture for approximately 20 s. As soon as a homogeneous liquid without air inclusions had formed, it was poured into the desired container and kept sealed in the refrigerator or freezer overnight. We included one frozen sample per tissue type to investigate the influence of temperature on the dielectric properties.

**Figure 1:**
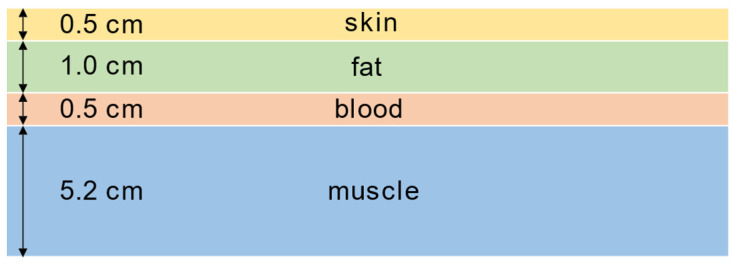
Schematic representation of the layered gelatin phantom.

After all samples were dielectrically characterized (see Section 2.2), the sample with the best dielectric properties was selected per tissue layer, and a human arm phantom was produced with these material compositions. We chose skin phantom 4, fat phantom 4, blood phantom 4 and muscle phantom 6 (see Section 3.2). For a realistic representation of the upper arm, the mixtures were poured into rectangular hard plastic boxes with a base area of 33 cm × 9 cm, as the average length of the *humerus* for men is 304.56 ± 14.16 mm [24]. The height varied depending on the tissue layer, similar to the anatomical tissue layer thickness of an average male arm [25]. We also considered the stability of the hydrogel layers when choosing a representative tissue layer thickness. Once the phantom tissues had cooled and hardened, they were removed from the boxes and stacked on top of each other. Figure 1 shows the positioning and the thicknesses of the individual layers.

### 2.2. Dielectric Characterization of Phantom Layers

The parallel plate method was used to measure the dielectric properties of the samples. This method was also used by Yu et al. [22], Toyoda et al. [18] and Kalra et al. [21] to validate gel-like tissue phantoms with regard to their dielectric properties. Apart from being widely used as standard measurement technique, the parallel plate setup has been shown to be highly appropriate, particularly for small samples and the low EMS frequency range [21,26]. 

**Figure 2:**
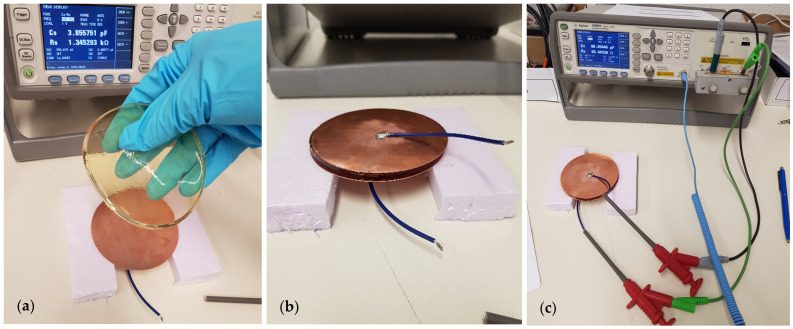
Measuring setup for dielectric validations of the samples. (**a**) Placing the phantom sample on the copper plate; (**b**) parallel plate setup with sandwiched sample; (**c**) measurement setup with parallel plate method and LCR meter.

The samples were measured between 20 and 60 min after removal from the refrigerator or between 60 and 90 min after removal from the freezer the day after fabrication. Hereby, we ensured that the refrigerated samples had reached room temperature and that the ice crystals on the frozen samples had thawed, while the samples were still cold. For the parallel plate setup, the sample was removed from the Petri dish and sandwiched between a pair of circular copper electrodes (diameter 850 mm, thickness 0.5 mm), placed on non-conductive polystyrene blocks (see Figure 2a,b). Subsequently, an LCR meter was connected to the measurement setup (see Figure 2c). We used the “E4980A precision LCR meter” (Keysight Technologies, Santa Rosa, CA, USA) to measure resistance R_s_ and capacitance C_s_ of the sample. 

**Figure 3:**
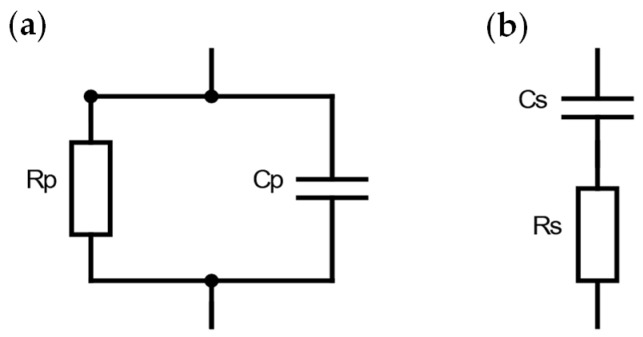
Possible equivalent circuits of the sample during dielectric measurement. (**a**) Parallel circuit and (**b**) series circuit of capacitance and resistance.

There are two ways in which the sample can be represented as an equivalent circuit during the measurement. It can be understood as a parallel connection of a capacitor with capacitance C_p_ and resistance R_p_ (see Figure 3a) or as a series connection of these components with capacitance C_s_ and resistance R_s_ (see Figure 3b). 

For impedances Z* < 100 kΩ, the serial equivalent circuit enables a more accurate measurement of capacitance [27]. Since the impedance of biological tissue lies in the range of a few hundred Ohms [28], we selected the serial equivalent circuit to measure the samples. If required, the serial measurement results could be converted into parallel values by calculation.

Before measuring the phantom samples, we conducted a short-circuit calibration of the measurement setup, including the cables, at 100 Hz. To validate the measurement setup, we first measured a 25% gelatin sample in the frequency range of 30 Hz to 100 kHz. Then, the results were compared with the same measurements from the literature [21], which also used the parallel plate method. Subsequently, we measured the tissue phantom samples at 20 Hz, 40 Hz, 60 Hz, 80 Hz and 100 Hz each. For every sample, we measured the parameters C_s_ and R_s_ at the respective frequencies. Each measurement was performed three times, and the measured values were then averaged. Finally, electrical conductivity σ and relative permittivity ε_r_ were calculated using the following Equations (1) and (2). Since cross-sectional area A and thickness d of the sample are known, relative permittivity ε_r_ can be determined as follows, using the measured capacitance C and the vacuum permittivity ε_0_ (8.854 × 10^−12^ F/m):

(1) $$\varepsilon_{r}=\frac{d\cdot C}{\varepsilon_{0}\cdot A}\;$$

Conductivity σ can be determined via resistance R, area A and thickness d of the sample as follows:

(2) $$\sigma =\frac{d}{R\cdot A}\;.$$

In Section 3.2, we compared the experimentally determined dielectric properties with tissue reference values from IT’IS database [23], which is based on Gabriel et al. [29], to evaluate the suitability of the phantoms for mimicking biological tissue.

### 2.3. EMS–EMG Measurement Setup

We performed EMS on the arm phantom while recording the voltage on the surface using an EMG sensor. The purpose of the measurement was to compare the resulting EMG waveform with the same measurement in humans to validate the phantom’s electrical transmission behavior. 

**Figure 4:**
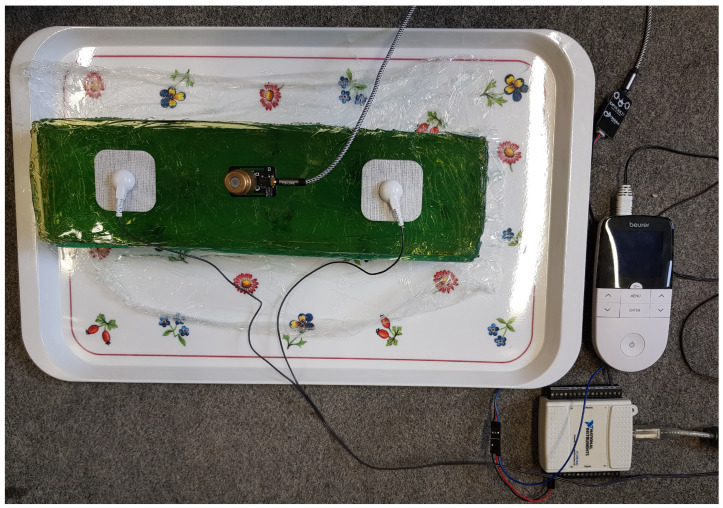
Measurement setup for electromyography (EMG) recordings during electromyostimulation (EMS) on the arm phantom.

The experimental setup included the EMS device “EM 49 Digital TENS/EMS” (Beurer, Ulm, Germany), EMS electrodes (45 × 45 mm, Beurer, Ulm, Germany) and an analog EMG sensor (“Gravity”, OYMotion, Shanghai, China) connected to a data acquisition device (“NI USB-6009”, National Instruments, Austin, TX, USA) and a 50 g weight that was placed on the EMG sensor to secure its position on the phantom. The data acquisition program was implemented in LabVIEW (National Instruments 2021). The EMS electrodes were attached to the proximal and distal ends of the arm phantom in the same way as in humans. EMG electrodes should be oriented parallel to the course of the muscle fiber and be positioned as centrally as possible on the belly of the muscle [30,31]. In the case of the phantom, this corresponds to a central position of the EMG electrode between both EMS electrodes. Figure 4 shows the experimental setup to perform EMS on the phantom and simultaneously record the resulting voltage signal on the surface via an EMG sensor. The applied stimulation signal was a biphasic rectangular pulse with a pulse width of 300 µs. We performed multiple measurements with varying frequencies (50 Hz, 100 Hz) at different intensity levels (9, 15, 23). The EMG signal was sampled at a frequency of 4 kHz.

For reference signals, the same measurements with the same electrode positioning were performed on a male human arm (*M. biceps brachii*, *n* = 1) in a relaxed position. The measurements were conducted in accordance with the Declaration of Helsinki and approved by the ethics committee of the TU Ilmenau (Reference No. 2024-03-196_FoA-Uhrha.1). Written informed consent was obtained from the volunteer. The only difference between the measurements on the phantom and the human arm was that the EMG sensor position was secured with skin tape on the subject’s arm instead of the weight used on the phantom. Additionally, the skin was disinfected before attaching the electrodes. We chose subthreshold stimulation intensities (level 9, 15, 23) to avoid visible muscle contractions in the EMG. The previously determined contraction threshold was an intensity level of 24. The subthreshold stimulation allows a comparison of the measured stimulation waveform between the arm phantom and the human arm, since no muscle reaction should be present in the signal.

## 3. Results

### 3.1. Validation of the Measurement Setup

**Figure 5:**
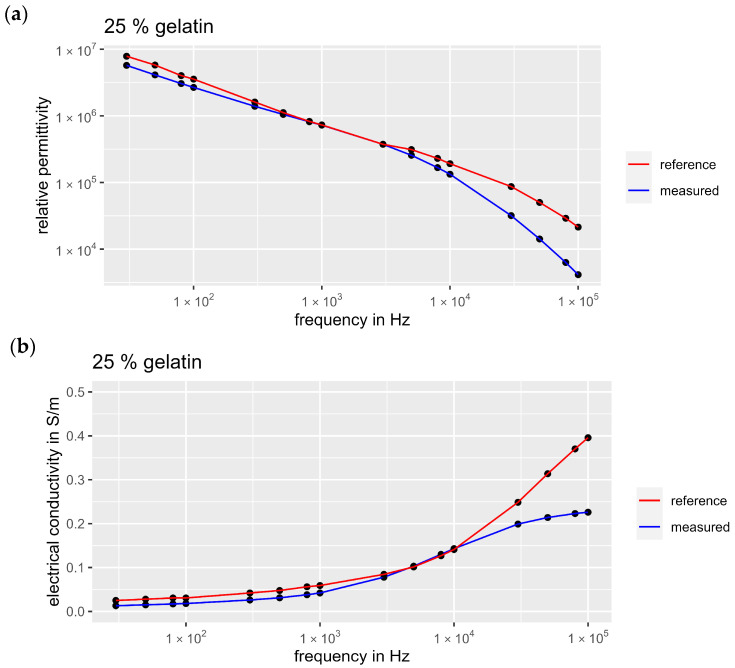
Comparison of the measured dielectric properties of a 25% gelatin sample with reference data from the study of Kalra et al. [21]: (**a**) relative permittivity, (**b**) electrical conductivity.

To validate the measurement setup, we reproduced the 25% gelatin phantom from the study by Kalra et al. [21] and compared our dielectric measurement data with the literature reference. As the original measurement data were not listed in [21], we used WebPlotDigitizer (automeris.io, version 5) to extract the values from the graphs. The extracted values and our dielectric measurement data can be found in the Supplementary Materials (Table S1). Figure 5 shows permittivity (a) and electrical conductivity (b) from the reference publication compared to our measurement results. The frequency curves qualitatively match well for both conductivity and permittivity, although especially the conductivity diverges above 1 × 10^4^ Hz. 

To quantitatively validate our measurement setup with the data from [21], we calculated the root mean square error (RMSE) between the frequency curves (R version 4.3.1). This resulted in an RMSE of 0.064 S/m for conductivity and 7.5 × 10^5^ for permittivity. Compared to the value range of the measurement data, these deviations are relatively small. When only considering the frequency range up to 1 × 10^4^ Hz, this results in an even lower RMSE of 0.012 S/m for conductivity. Consequently, the measurements are consistent, especially in the low-frequency range relevant for EMS applications. Therefore, the measurement setup can be considered validated.

### 3.2. Dielectric Properties of the Tissue Phantoms

**Figure 6:**
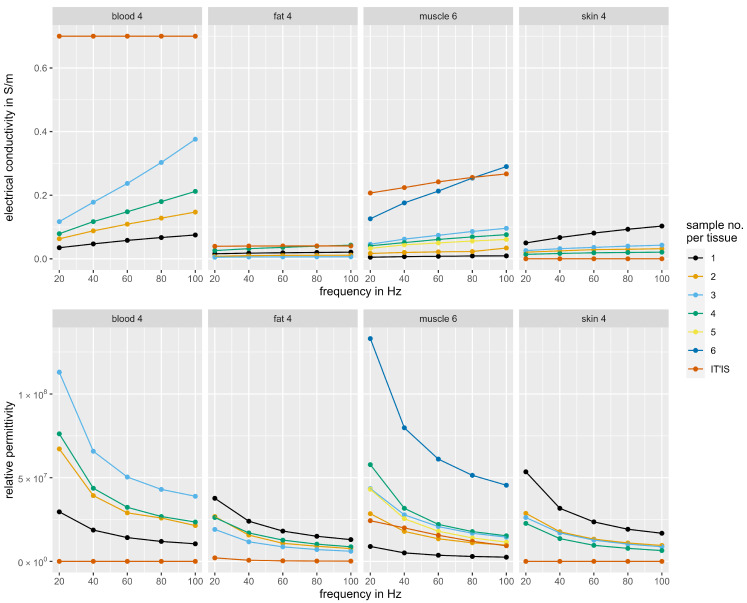
Dielectric properties of different tissue phantoms compared to reference values of biological tissue from IT’IS database [23].

In this section, the experimentally determined dielectric properties of the phantoms are compared with those from the IT’IS Foundation reference database [23]. Figure 6 shows the measured electrical conductivity and relative permittivity of the different tissue phantom samples with varying frequencies. It can be observed that the phantom samples with the best approximation to biological tissue either mimic conductivity or permittivity, but not both at the same time. This can be clearly seen in the example of blood phantom 1, as it simulates the permittivity of blood best, but at the same time it represents the conductivity worst. This opposite trend was also observed for the other tissue types. For the fat and skin phantoms, we included two samples with identical mixing ratios where one sample was frozen overnight. For the skin phantom, we found that the permittivity and conductivity in the cold sample (4) decreased compared to the room temperature sample (3). The fat phantom showed hardly any changes between the cold (2) and warm samples (3). Mixing ratios with high salt content led to an increase in conductivity and permittivity (blood phantom, low salt content: sample 1, higher salt content: sample 2). In contrast, high oil content, as for the fat phantoms, led to reduced permittivity and conductivity. 

In order to select the sample with the best dielectric properties for each tissue type, a compromise had to be found between mimicking permittivity and electrical conductivity. For this purpose, the relative deviation from the biological reference was calculated for each sample. This was carried out separately for permittivity and conductivity, and then the samples were ranked per tissue type and dielectric property based on their relative deviation. Within a tissue type, the mean value of the ranks for permittivity and conductivity was then calculated for each sample. For each tissue type, the sample with the best average ranking was finally selected for the fabrication of the arm phantom. The measured values for electrical conductivity and relative permittivity of the selected phantom samples are shown in Table 2 in comparison with biological reference data. The measurement data of all samples can be found in the Supplementary Materials (Table S2).

**Figure 7:**
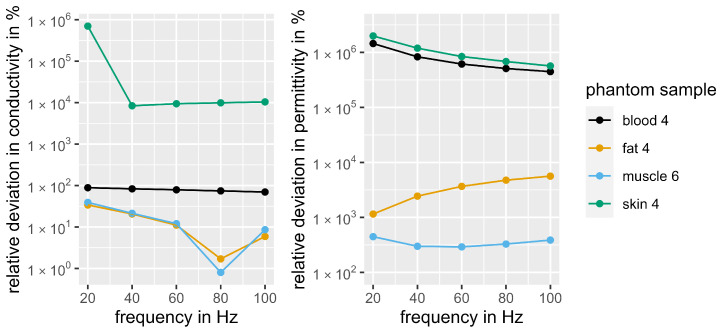
Relative deviations in conductivity and permittivity of the selected phantom samples over the course of frequency.

On average, blood phantom 4 achieved the best imitation of the dielectric properties of blood. It also offered better mechanical stability than blood phantom 3, which achieved the best conductivity but the worst permittivity. Figure 7 shows the relative deviations in permittivity and conductivity of the selected phantom samples over the course of frequency. The relative deviation in conductivity for blood phantom 4 ranges from 69.7% (100 Hz) to 88.7% (20 Hz). Fat phantom 4 was chosen to mimic adipose tissue. It achieves a very good approximation of the conductivity with a deviation of 1.7% (80 Hz), while the permittivity shows higher deviations (593.8% at 20 Hz). The mixing ratio of muscle sample 6 was selected for mimicking muscle tissue. Just like the fat phantom, it shows a good approximation of the conductivity (0.8% deviation at 80 Hz), whereas the permittivity deviates more strongly from the biological reference (328.3% at 80 Hz). Regarding the deviations in the course of frequency, there are no consistent behaviors or trends visible, apart from the clear minima in deviations of conductivity for the muscle and fat layer at 80 Hz. The other frequency profiles remain mostly constant, except for a noticeable maximum deviation in conductivity at 20 Hz for the skin layer and a slight upwards trend in permittivity deviations for the fat sample. Consequently, the phantom represents the dielectric properties of biological tissue best at 80 Hz and worst at 20 Hz.

Overall, out of all tissue types, the best approximation to biological tissue was achieved with the muscle phantom. In contrast, the dielectric replica of skin was the furthest away from the biological reference. Since both permittivity and conductivity of skin are very low, there were very high relative deviations from the phantom samples. The best sample was skin phantom 4, which achieved an average deviation for permittivity and conductivity of 288,483.3% (100 Hz). This corresponds to an absolute deviation in conductivity of 0.0208 S/m and in relative permittivity of 6.46 × 10^6^.

### 3.3. EMS–EMG Measurement Results 

To evaluate the performance of the arm phantom during EMS, we conducted simultaneous EMG measurements and compared the resulting signals with the same measurements on a human arm. To compare the tissue composition between phantom and human, we calculated the subject’s fat layer thickness. Using the equation for the fatty layer thickness of the arm presented in [32], we calculated a thickness of 4.1 mm, based on the subject’s gender (male), age (27) and body mass index (21.5). The thickness of the phantom’s fat layer was 10 mm. 

**Figure 8:**
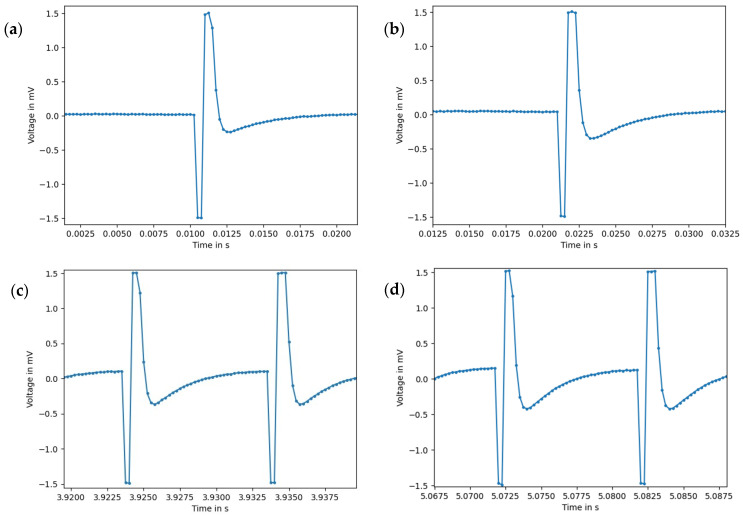
EMG signal section during electrical stimulation on the arm phantom vs. on a human arm: (**a**) phantom, 50 Hz, intensity level 15; (**b**) human, 50 Hz, intensity level 15; (**c**) phantom, 100 Hz, intensity level 23; (**d**) human, 100 Hz, intensity level 23. Pulse width: 300 µs, sampling rate: 4 kHz.

To compare the electrical transmission behavior between the phantom and human arm, we carried out EMG measurements with different stimulation frequencies and intensities. Figure 8 shows two measurements of different frequencies recorded on the phantom versus on a human arm. We found that the signal shape is generally identical between phantom and biological reference. Each signal section consists of a biphasic rectangular stimulation pulse followed by a slight overshoot in the opposite direction. We recorded an increase in the amplitude of the overshoot with increasing pulse intensity in both the phantom and the human arm. The amplitude of the stimulation pulse is cut off by the sensor range (−1.5 mV to 1.5 mV), and is therefore not displayed completely. 

For a quantitative comparison between the measurements on the phantom and human arm, we calculated the root mean square (RMS) amplitude for each stimulation setting. Table 3 shows an overview of the RMS amplitudes and relative deviations between phantom and human. It is noticeable that the RMS amplitude increases with increasing stimulation intensity and frequency in both phantom and human. The RMS amplitudes of the same stimulation setting hardly differ between phantom and human, which is consistent with the similar signal curves in Figure 8. The relative deviations range from 1.9% to a maximum of 9.5%, whereby lower amplitudes were measured for the phantom than for the human arm. This is not surprising, as the poorly conductive fat layer was thicker in the phantom than the human arm.

Overall, the comparative EMS–EMG measurements between phantom and human, with similar signal shapes and deviations in RMS amplitude of less than 10%, show that the phantom has an electrical transmission behavior comparable to human tissue.

## 4. Discussion

Tissue phantoms are frequently used in many areas of medical research. However, there is a lack of research for dielectric tissue phantoms in the low-frequency range, and especially for EMS applications. In our study, we fabricated a human arm phantom for EMS applications with the tissue layers of skin, fat, blood and muscle. We used common materials from the literature and extensively tested the dielectric properties in the low-frequency range (20–100 Hz). Despite the partly high deviations in the dielectric properties from biological tissue, the phantom reproduces the electrical transmission behavior qualitatively and quantitatively well. 

Since we examined various phantoms with different material compositions regarding their permittivity and conductivity (Figure 6), we were able to analyze the influence of additives and temperature on the dielectric properties. As expected, an increased salt content leads to an increase in conductivity, as more charge carriers are present in the sample (Figure 6, blood phantom, low salt content: sample 1, higher salt content: sample 2). It is also not surprising that an increased oil content leads to a reduction in permittivity and conductivity, as the proportion of non-polar molecules in the sample increases (Figure 6, fat phantoms). Non-polar molecules form neither a positive nor a negative pole, so the electrical charges in the molecules are evenly distributed [28]. While water, with its polar molecules, supports the spread of electric fields, this is not the case with oil. For the blood phantom, we observed that a simultaneous increase in the salt and oil content leads to an overall increase in conductivity and permittivity. This observation indicates that the addition of further charge carriers has a greater effect on the dielectric properties than the addition of non-polar molecules. 

Figure 6 shows that the cold skin phantom (4) frozen overnight has a lower permittivity and a lower conductivity compared to the unfrozen sample (3). Storing the samples at negative temperatures causes the water molecules to arrange themselves into a lattice [33]. The sample is frozen and the mobility of electrical charge carriers is severely restricted. Not only does the electrical conductivity depend on the mobility of charge carriers, it also depends on the capacitance. If charge carriers are immobile in the frozen state or less mobile in the cold thawed state, no or only less charge can be stored, which leads to a small capacitance and a high specific resistance. In the case of the fat phantom, the temperature affected the tissue phantom less than the case with the other tissue types, owing to their higher water content. Freezing the fat phantom overnight had surprisingly little effect on the properties measured (Figure 6, fat phantom, cold: sample 2, room temperature: sample 3). One possible reason for this is that oil molecules are non-polar compared to water molecules, and therefore have no hydrogen bonds and consequently no crystal structure forms during freezing [33]. Based on the determined influences of the additives and the temperature, future research may focus on modifying the mixing ratios in order to obtain better approximations to the biological reference. Regardless of the fact that freezing the samples did not lead to the desired results, we advise against varying the sample temperature in order to adjust the dielectric properties. The reason for this is the impracticality of deviations from room temperature for further EMS examinations on the phantom. Then, the phantom would have to maintain a specific temperature for the entire duration of the examination, which would involve a lot of effort. For this practical reason, we did not make any further temperature-dependent considerations and optimized our samples for studies at room temperature. 

In general, we found that it is difficult to identify mixing ratios that have both good permittivity and good conductivity compared to the biological reference (Figure 6). This can be exemplarily seen with blood phantom 1, as it simulates the permittivity of blood best, but at the same time represents the conductivity worst. A compromise had to be found between the two physical quantities, resulting in high deviations in permittivity for some frequencies and phantom types. Despite this, the comparison of the EMG measurements on the phantom with those on a human arm showed that the phantom has a comparable electrical transmission behavior of the EMS pulse (Figure 8 and Table 3). Therefore, we assume that the electrical conductivity has a larger impact on the transmission of the EMS pulse than the relative permittivity. The observed increase in the amplitude of the overshoot with rising intensity is presumably due to charge equalization after the stimulation pulse, which increases with higher stimulation voltage. Therefore, the phantom appears to be suitable for EMS investigations. However, further research should investigate how precisely the dielectric properties must be reproduced in order to measure voltages reliably. In this context, it should be investigated why signal shape and RMS amplitude between phantom and biological reference match so well, although there are some deviations in the dielectric properties. 

Since we based our mixing ratios on research described in the literature, it is interesting to compare the measurement results. For the validation of the measurement setup and for some samples of the fat and skin phantoms, we followed Kalra et al. [21], who also used the parallel plate method to measure dielectric properties. The comparison of our measurements with the literature reference is presented in Figure 5. Since no numerical data were listed in the reference publication, we extracted the data from the graphs by using WebPlotDigitizer. As the web-based tool requires manual setting of the calibration and measuring points for digitizing the graphs, deviations from the actual measured values may occur. However, study results show that WebPlotDigitizer is a reliable and valid method for obtaining data from plots with a high intercoder reliability (over 90% proportional agreement) [34]. 

We were able to reproduce the frequency-dependent course of permittivity and conductivity for the 25% gelatin phantom with minor deviations. Therefore, the measurement setup was considered validated, as the deviations fell within the high-frequency range irrelevant for EMS purposes. The marginal differences between the measurements could be attributed to electrode polarization (EP), which was mathematically corrected in the study of Kalra et al. [21], but not considered in our data. EP describes the formation of unwanted electrical double layers on the interface of the sample and the electrodes, resulting in an increase in the impedance measured [22]. The influence of EP on permittivity generally is stronger in the low-frequency range than in the high-frequency range [22,35]. However, this correlation cannot be seen when comparing our results with the study of Kalra et al. [21]. The deviations in the permittivity are not higher in the low-frequency range than in the high-frequency range. This indicates that EP did not substantially influence our measurement results, and that the deviations probably arose from other causes. In general, it can be stated that it is difficult to mimic skin with a water-based gelatin model, as the phantom should have low conductivity and low permittivity. A more insulating material, such as silicone, would probably be more suitable and should be considered in future research. 

For the muscle phantom, we followed the mixing ratios of Yu et al. [22], who considered the frequency range of 10 Hz to 100 Hz and also carried out dielectric measurements using the parallel plate method. In contrast to our measurements, they compensated the EP mathematically, although we still obtained qualitatively comparable results for the frequency range measured. As no specific values or deviations to biological tissue are given in the publication, no quantitative statements about the consistency of our data can be made. Khorshid et al. [17] and Costanzo et al. [16] produced hydrogel tissue phantoms with similar material compositions to ours, but achieved smaller deviations (max. 10% for permittivity and 36% for conductivity) than biological tissue. However, their measurements were conducted for a considerably higher frequency range (100 kHz–100 MHz, respectively, 500 MHz–5 GHz) than ours (20–100 Hz). Another reason for why they could report better approximations is that they considered the deviations for conductivity and permittivity separately and only for specific frequencies. In contrast, we tried to achieve the best possible approximations for both parameters simultaneously and for the whole frequency range measured. 

Our results show that hydrogel phantoms with low-cost ingredients such as oil, salt and dishwashing detergent exhibit greater deviations in permittivity and conductivity in the low-frequency range than comparable phantoms in the high-frequency range. However, according to the current state of the art, no better approximations can be expected for the low-frequency range. In the research of Kalra et al. [21], the permittivity of the skin phantom (measured value approx. 10 × 10^7^) deviates by a factor on the order of 10 × 10^4^ from the permittivity of dry skin (measured value approx. 10 × 10^3^). Apart from the frequency range, the good approximations reported in the literature are also due to the fact that permittivity and conductivity were not considered simultaneously, and good approximations were only achieved for individual frequencies. In addition, in some cases, no quantitative deviations from the biological reference were calculated, but only the qualitative data were compared. The novelty of our research lies in filling the gap of dielectric tissue phantoms for EMS applications by presenting a multilayer arm phantom that tries to mimic both relative permittivity and electrical conductivity for all relevant frequencies (20–100 Hz), using low-cost ingredients and considering the temperature of the samples.

A limitation of our research is the simplified phantom geometry, as we chose a rectangular layered arm phantom instead of a cylindrical arrangement. This was due to the mechanical fragility of the hydrogel layers, which would have made it difficult to bend them. We also considered only the upper half of the arm without bone, because in future investigations we are mainly interested in the effects inside the muscle together with the influences of the overlying structures. Another limitation of the hydrogel phantom is its limited durability due to the perishable ingredients. However, its fabrication is fast and inexpensive, and thus the phantom can be easily reproduced when needed. The last important aspect when comparing our data with the tissue references is the fact that Gabriel et al. [29] used a different measuring method and a different measuring device. The measuring method can considerably influence the measured dielectric quantities, which can lead to measurement deviations [36]. 

Considering the comparable pulse form and low RMS deviations between phantom and subject, the use of the arm phantom can be recommended for further EMS investigations. The phantom is particularly suitable for 80 Hz, as this is where the deviations in conductivity to the biological reference reach their minimum (1.7% for fat, 0.8% for muscle). However, the results should be interpreted with caution, as the permittivity partly shows large deviations from biological tissue. Our dielectric arm phantom enables future investigations of the voltage distribution in the tissue during EMS. This allows the examination of penetration depth and voltage level at different points depending on the electrode size, electrode position, tissue composition and stimulation parameters. Not only surface electrodes, but also needle electrodes can be used to tap the voltage in deeper tissue layers. This knowledge could make an important contribution in increasing the efficacy and safety of EMS, since the relevant parameters could be specifically controlled so that the muscles are adequately stimulated. 

## 5. Conclusions

Within the scope of this research, a multilayered upper arm phantom with skin, fat, blood and muscle tissue types was developed for the application purpose of EMS. The aim of the research was to mimic the dielectric properties of biological tissue so that future investigations on the voltage distribution during EMS could be performed on the phantom. We used low-cost materials, such as gelatin, distilled water, iodized salt, dishwashing detergent and sunflower oil, in different mixing ratios to produce a total of 18 tissue samples. We dielectrically characterized the samples using the parallel plate method and found that skin phantom 4, fat phantom 4, blood phantom 4 and muscle phantom 6 best replicate the dielectric properties of biological tissue (deviations ranging from 0.8% to more than 100%). Especially at 80 Hz, the phantom achieved the best approximations to the conductivity of biological tissue (1.7% deviation for fat, 0.8% for muscle). Despite the partly high deviations in permittivity, the voltage signals measured on the phantom and the human arm during EMS were qualitatively and quantitatively consistent, with deviations in RMS amplitude ranging from 1.9% to 9.5%. An important finding is that mimicking the permittivity and electrical conductivity at the same time is more demanding in the low-frequency range (20–100 Hz) than in the high-frequency range (MHz–GHz). This paper makes a valuable contribution in the field of dielectric tissue phantoms, as it proposes a simple and low-cost arm phantom considering both permittivity and electrical conductivity at the same time for all measured frequencies in the EMS frequency range. Looking ahead, our phantom can be used for primary EMS investigations, but future research should focus on further reducing the permittivity without changing the conductivity of the samples. 

## Figures and Tables

**Table 1:** Material concentrations of the different tissue phantoms.

Phantom Type	Sample Number	Reference	Gelatin		Distilled Water		Sunflower Oil		Dishwashing Detergent		Iodized Salt	
			g	% (*w*/*w*)	g	% (*w*/*w*)	g	% (*w*/*w*)	g	% (*w*/*w*)	g	% (*w*/*w*)
Skin	1	[16]	3.4	9.6	19.4	54.6	10.9	30.7	1.0	2.8	0.8	2.3
	2	[16]	3.5	9.2	15.3	40.6	15.0	39.8	3.9	10.3	-	-
	3	[21]	10 *	25	30	75	-	-	-	-	-	-
	4 **	[21]	10 *	25	30	75	-	-	-	-	-	-
Fat	1	[16]	2.2	3.8	8.2	14.2	45.5	78.9	1.8	3.1	-	-
	2 **	[16]	2.2	3.9	8.2	14.4	45.0	79.2	1.4	2.5	-	-
	3	[16]	2.2	3.9	8.2	14.4	45.0	79.2	1.4	2.5	-	-
	4	[21]	10 *	25	30	75	-	-	-	-	-	-
Blood	1	[16]	3.3	10.3	21.8	68.3	2.4	7.5	4.0	12.5	0.4	1.3
	2	[16]	3.3	9.9	16.9	51.1	6.3	19.0	4.6	13.9	2.0	6.0
	3 **	[16]	5.0	13.9	17.0	47.2	6.0	16.7	4.0	11.1	4.0	11.1
	4	-	5.0	13.2	30.0	78.9	-	-	3.0	7.9	-	-
Muscle	1	[16]	2.6	5.5	14.5	30.9	28.4	60.4	1.5	3.2	-	-
	2	[16]	2.6	5.5	14.5	30.9	28.4	60.4	1.5	3.2	-	-
	3	[22]	5.3	14.9	30.0	84.3	-	-	0.3	0.8	-	-
	4	[16]	2.6	5.5	14.3	30.1	28.2	59.4	1.5	3.2	0.9	1.9
	5 **	[22]	5.3	14.5	30.0	81.9	-	-	1.3	3.6	-	-
	6	[22]	5.3	14.8	30.0	83.8	-	-	0.5	1.4	-	-

* Usage of gelatin powder. ** Stored in the freezer.

**Table 2:** Electrical conductivities and relative permittivities of the selected phantom samples compared with biological tissues at different frequencies.

Frequency/Hz		20	40	60	80	100
**Electrical Conductivity/S/m**	Blood Phantom 4	7.90 × 10^−2^	1.17 × 10^−1^	1.48 × 10^−1^	1.80 × 10^−1^	2.12 × 10^−1^
	Blood *	7.00 × 10^−1^	7.00 × 10^−1^	7.00 × 10^−1^	7.00 × 10^−1^	7.00 × 10^−1^
	Fat Phantom 4	2.60 × 10^−2^	3.20 × 10^−2^	3.60 × 10^−2^	3.99 × 10^−2^	4.30 × 10^−2^
	Fat *	3.94 × 10^−2^	4.03 × 10^−2^	4.05 × 10^−2^	4.06 × 10^−2^	4.06 × 10^−2^
	Muscle Phantom 6	1.26 × 10^−1^	1.76 × 10^−1^	2.13 × 10^−1^	2.54 × 10^−1^	2.90 × 10^−1^
	Muscle *	2.07 × 10^−1^	2.24 × 10^−1^	2.42 × 10^−1^	2.56 × 10^−1^	2.67 × 10^−1^
	Skin Phantom 4	140 × 10^−2^	1.70 × 10^−2^	1.90 × 10^−2^	2.00 × 10^−2^	2.10 × 10^−2^
	Skin *	2.00 × 10^−4^	2.00 × 10^−4^	2.00 × 10^−4^	2.00 × 10^−4^	2.00 × 10^−4^
**Relative Permittivity**	Blood Phantom 4	7.62 × 10^7^	4.37 × 10^7^	3.23 × 10^7^	2.68 × 10^7^	2.35 × 10^7^
	Blood *	5.26 × 10^3^	5.26 × 10^3^	5.26 × 10^3^	5.26 × 10^3^	5.26 × 10^3^
	Fat Phantom 4	2.62 × 10^7^	1.70 × 10^7^	1.27 × 10^7^	1.03 × 10^7^	8.71 × 10^6^
	Fat *	2.09 × 10^6^	6.69 × 10^5^	3.37 × 10^5^	2.12 × 10^5^	1.52 × 10^5^
	Muscle Phantom 6	1.33 × 10^8^	7.98 × 10^7^	6.11 × 10^7^	5.14 × 10^7^	4.55 × 10^7^
	Muscle *	2.43 × 10^7^	2.00 × 10^7^	1.56 × 10^7^	1.20 × 10^7^	9.33 × 10^6^
	Skin Phantom 4	2.27 × 10^7^	1.36 × 10^7^	9.58 × 10^6^	7.78 × 10^6^	6.46 × 10^6^
	Skin *	1.14 × 10^3^	1.14 × 10^3^	1.14 × 10^3^	1.14 × 10^3^	1.14 × 10^3^

* Reference data for biological tissue from IT’IS Foundation database [23].

**Table 3:** Root mean square (RMS) amplitudes measured on phantom and human arm at different stimulation settings with relative deviations between phantom and human for each measurement. I = intensity level.

	RMS Amplitude in mV		
EMS Parameters	Phantom	Human	Relative Deviation in %
50 Hz, I 9	0.35	0.36	2.8
50 Hz, I 15	0.36	0.37	2.7
50 Hz, I 23	0.38	0.42	9.5
100 Hz, I 23	0.53	0.54	1.9

## Data Availability

The original contributions presented in the study are included in the Supplementary Materials; further inquiries can be directed to the corresponding author.

## Supplementary Materials

The following supporting information can be downloaded at: https://www.mdpi.com/article/10.3390/bioengineering11070724/s1, Table S1: Data of the measurement setup validation, Table S2: Dielectric measurement data of the tissue phantoms.
